# Using think aloud with female adolescents to validate psychological well- and ill-being self-report measures

**DOI:** 10.1371/journal.pmen.0000551

**Published:** 2026-02-17

**Authors:** Sophie C. M. Chatwin, Rebbeca M. Pearson, Haridhan Goswami, Paul R. Appleton

**Affiliations:** 1 Department of Sport and Exercise Sciences, Manchester Metropolitan University, Manchester, United Kingdom; 2 Institute of Sport, Manchester Metropolitan University, Manchester, United Kingdom; 3 Department of Psychology, Manchester Metropolitan University, Manchester, United Kingdom; 4 Department of Sociology, Manchester Metropolitan University, Manchester, United Kingdom; National University of Singapore, SINGAPORE

## Abstract

Many existing measures of psychological well- and ill-being are used with young people without testing contemporary adolescent understanding of them. The purpose of this study was to use think aloud interviews to test adolescent understanding of three existing, and one newly created psychological well- or ill-being measures. An initial sample of 40 female participants aged 13–14 years took part in the study. Problematic items were identified based on thematic analysis of adolescent feedback. One item of the Lethargy Scale and one item of the Subjective Vitality Scale were adapted following integration of think aloud findings. The results also indicated that the Brief Serenity Scale was unsuitable for the participants. Subsequently, a second sample of 57 female participants aged 13–14 years completed the think aloud protocol with an alternative measure of serenity, the Child Serenity Scale, and thematic analysis revealed no items were problematic. A 24-item psychological well- and ill-being self-report measure was consequently proposed for use in future research.

## Introduction

Globally, 14% of adolescents report having a mental health disorder such as anxiety, depression, eating disorders and addictive behaviours [1]. National Health Service (NHS) data shows one in five children and young people in England aged eight to 25 had a probable mental health disorder in 2023, which is worse since the COVID-19 pandemic [2]. Moreover, the Children’s Society found 11% of 10- to 17-year-olds in the United Kingdom (UK) had low well-being, determined by low life satisfaction scores which were almost half than the European average [3]. Failing to address these issues in adolescence has consequences that stem into adulthood; 50% of adult mental health disorders onset by the age of 14, and ill-being is associated with an increased likelihood of impaired social relationships, substance misuse, and criminal behaviour [4].

Defining psychological well-being is complex because there are numerous conceptualisations, and no universally accepted definition. There is one unanimous theme however, and that is psychological well-being is defined as hedonic or eudemonic [5]. The hedonic approach defines psychological well-being in terms of life satisfaction and subjective happiness. The eudemonic approach focuses on optimal functioning [5]. Ryan and Deci [6] and Ryff [7] suggest due to its multidimensional nature, well-being should include both hedonic and eudemonic aspects. Ill-being has been relatively neglected compared to well-being in literature [8]. Yet again, there is no universally accepted definition of psychological ill-being. Many studies imply ill-being is the opposite of well-being or fail to provide a definition beyond “e.g., depression, stress” [9]. More recently, ill-being has been defined as “unpleasant feelings or emotions that impact the level of functioning” [10], and “negative psychological states” [11]. There is also a debate whether well- and ill-being are independent dimensions, or whether they sit at opposite ends of a bipolar continuum. The continuum theory suggests individuals with higher levels of ill-being would have lower levels of well-being and vice versa [12]. The independence theory suggests higher levels of ill-being would not necessarily be associated with lower levels of well-being and vice versa [12]. Bradburn’s [13] research into independence versus bipolarity discovered the association between positive and negative affect was not strong, despite being inversely correlated. Therefore, scoring high for one construct does not predict scoring for the other construct. The present study will take the view that psychological well- and ill-being are independent, and therefore need to be measured simultaneously but independently.

A criticism of well- and ill-being theories is that they were developed by adults, often for adults [14], but are regularly applied to young people without consideration of their relevance and applicability. Taking two hedonic theories, subjective well-being [15] and quality of life [16] as examples, well-being in the work domain, which is central to these theories, is not relatable for all stages of adolescence. It could be argued that these conceptualisations, and related measures based on these conceptualisations, may not accurately capture an adolescent’s experience of psychological well- and ill-being. To overcome the adult centrism often seen in previous research, some studies have involved adolescents when conceptualising well-being [17,18], but less attention has been paid to ill-being. In one study however, Bracey [19] asked a sample of young people in the UK to write down words they associated with well- and ill-being, and where they felt these feelings (head, body, and/or heart). Younger participants (aged 7–11 years) defined well- and ill-being as unidimensional, singularly occurring feelings. Older participants (aged 12–15 years) had a greater sense of their complexity, describing well-being as both hedonic and eudemonic (“you’d be happy, you’d be healthy…content with…life”), and ill-being as multidimensional (“scared”, “upset”, “you feel bad”) [19]. Bracey’s involvement of adolescents, and consideration of both hedonic and eudemonic perspectives, arguably produced more appropriate definitions for adolescents than existing adult definitions. Focusing on the psychological domain of well- and ill-being, Bracey’s [19] definitions are adopted in this study. Psychological well-being is having life satisfaction, happiness, and optimal functioning determined by positive affect, vitality, and serenity. Psychological ill-being is negative emotional states and undesirable psychological conditions determined by negative affect and lethargy. Participants in Bracey’s [19] work also identified hyperactivity as a facet of ill-being, however in the current study, it was decided to remove this from the definition due to its connotations with neurodivergent conditions such as attention deficit hyperactivity disorder (ADHD). Additionally looking at existing hyperactivity measures, they are often intended for clinical practice, or items reflect an outcome of ill-being rather than a facet of ill-being (e.g., “I was easily distracted, I found it difficult to concentrate” from the Strengths and Difficulties Questionnaire [20]).

A large body of research has examined young people’s psychological well- and ill-being utilising a variety of measures (e.g., Scale of Positive and Negative Experience (SPANE) [21]; Warwick-Edinburgh Mental Well-Being Scale (WEMWBS) [5]; Children’s Society Index of Children’s Subjective Wellbeing (CSICSW)) [22]. Although previous measures are well-validated, they have limitations. They often only consider either eudemonic or hedonic well- (but not ill-) being; as noted above, several measures were developed by adults, for adults, but subsequently used with children/adolescents; and the content validity of these measures is rarely tested. The COnsensus-based Standards for the selection of health Measurement INstruments (COSMIN) can facilitate the assessment of the content validity of self-report measures [23]. According to COSMIN guidelines, relevance, comprehensiveness, and comprehensibility of items from the perspective of the participants and the professional (i.e., researcher) need to be established for a self-report measure to have good content validity. With existing measures, COSMIN guidelines are not always met. For example, regarding the WEMWBS [5] the comprehensibility of items was poor with young participants, as items caused confusion, were misinterpreted, or participants were hesitant to answer [4]. This could be due to the measure stemming from adult conceptualisations of well-being, and/or the reading age not being suitable for younger people. Some measures have been developed from the perspective of adolescents albeit with a limited definition of what constitutes well- and ill-being (i.e., according to COSMIN guidelines, more relevant to the participants but not comprehensive in terms of content). Rees et al. [22] for example, chose to define well-being as unidimensional in terms of quality of life and all five items of the CSICSW include “life”, such as “my life is going well”, which does not capture the multidimensional nature of psychological well-being.

Several specific measures exist which capture the various facets of psychological well- and ill-being proposed by Bracey [19]. For example, the Positive and Negative Affect Scale-Child (PANAS-C) [24,25] measures adolescent positive and negative affect which are defined as “general emotional states which have a positive and negative valence and content, respectively” [19]. The Subjective Vitality Scale (SVS) [26] measures vitality which is defined by Bracey as “a positive state of activation marked by dynamic liveliness and energy” [19]. Although developed for adults, a 5-item version of SVS has been adopted in research with children [27]. Less research attention has been paid to serenity (“a positive state of deactivation marked by composed tranquillity and inner peace” [19]) as an indicator of psychological well-being. Studies that have targeted serenity in adults have employed the 65-item Serenity Scale (SS) [28] or the 22-item Brief Serenity Scale (BSS) [29], although no study has adopted either scale when measuring serenity in young people. With regards to lethargy, defined as “a negative state of deactivation marked by apathetic lethargy and ennui” [19], to date there are no measures that fit this definition. Some researchers have used single items such as “did you feel more tired yesterday or today than you usually do?” (Memorial Symptom Assessment Scale (MSAS 7–12) [30], and “how often do you feel tired when you go to school in the morning?” [31]. However, these items are a better measure of tiredness, rather than lethargy. Another issue is that studies often use lethargy interchangeably with fatigue;. whereas lethargy is a temporary “negative state of deactivation” [19], fatigue is extreme “tiredness resulting from physical or mental exertion or illness” [32]. This suggests measures of fatigue are not a suitable replacement for measures of lethargy. Therefore, a measure which focuses solely on lethargy as defined within Bracey’s [19] work and as experienced by young people is needed.

### The present study

Previous research has utilised a variety of scales and questionnaires to capture indicators of young people’s psychological well- and ill-being. Although many scales have acceptable psychometrics, key limitations are discussed above. Moreover, to date, no scale exists that we are aware of that captures the multidimensional nature of psychological well- and ill-being as experienced by young people (in [19]). To begin to address these limitations, the present study used think aloud methods [33] with female adolescents to examine the content validity of four questionnaires that align with the definitions of well- and ill-being developed by Bracey [19]: Positive and Negative Affect Scale-Child (PANAS-C) [25], Subjective Vitality Scale (SVS) [26], and the Brief Serenity Scale (BSS) [29]. As mentioned above, as there are no suitable measures for Bracey’s [19] definition of lethargy, four items were developed in this study (see methods for more detail).

In this study, the focus was specifically on adolescent girls. Girls are more susceptible to lower levels of well-being than boys of the same age [34]. Deighton et al. [34] compared levels of well-being in a sample of 10,889 British adolescents each year for three years (11–14 years old). The results showed significant gender differences as emotional difficulties were higher for girls, behavioural difficulties were higher for boys (although girls’ scores were increasing each year) and subjective well-being was lower for girls [34]. It is clear female adolescents, especially aged around 14 years, are a particularly vulnerable group to these health risks and were therefore the target sample in this study.

## Method

### Ethics statement

Prior to the study commencing, ethical approval was obtained from the Faculty of Science and Engineering Ethics and Governance Committee at Manchester Metropolitan University, approval number 58960. As participants were children, written parent/guardian consent was obtained first, then the participants’ written assent was obtained. Participants’ identifying information were removed from the transcripts to assure confidentiality and anonymity. Participants were referred to according to identification codes, whereby each unique participant (P) was numbered (e.g., P1).

### Transparency and openness

This study’s design and its analysis were not preregistered. All data, analysis code, and research materials are available upon request from the first author. Data were analysed using IBM SPSS Statistics version 29.0 [35].

### Participants

Participants (N = 40) were female adolescents from the UK aged 13–14 years old (M = 13.3; SD = 0.46). Data collection started on 05/02/2024 and ended on 12/04/2024. Participants were recruited via emails to gatekeepers at schools (i.e., Headteachers) and sports clubs (i.e., coaches). Potential participants were provided with a: (1) parent/guardian information sheet, (2) parent/guardian consent form, (3) participant information sheet, and (4) participant assent form via onlinesurveys.ac.uk. The information sheets stated that participation was voluntary, interviews would be audio recorded on Microsoft Teams, any quotes included in the analysis would be anonymous, and that participants were free to withdraw from the study at any point. Participants could opt in to a prize draw for a £20 Amazon voucher. Participants were recruited from two schools and two sports clubs in the West Midlands in the UK. Using the Index of Multiple Deprivation tool [36], these locations are 89–92% more deprived than other neighbourhoods in England.

### Procedure

In the think aloud method, participants are asked to verbalise their thoughts while completing a task, such as a questionnaire, to reveal their thought processes [33]. There are two variations of think aloud. In concurrent reporting, participants naturally express any thoughts they have about the task without attempting to explain them [33]. In immediate retrospective reporting, the researcher asks the participant questions immediately after the task to elicit additional thoughts [33]. Previous studies have shown that think aloud interviews are useful for identifying usability issues with questionnaires, such as stumbling, rereading and misinterpreting items [37,38]. This study employed immediate retrospective reporting, enabling the researcher to clarify any items that participants may not understand and to gather their suggestions on how to reword problematic items.

Think aloud interviews were conducted either online using Microsoft Teams (N = 1) or in a quiet room at a location convenient to the participant such as their school or sports club (N = 39). All participants completed the questionnaires using onlinesurveys.co.uk on a laptop. At the beginning of the interview, participants completed a short questionnaire to provide demographic information. Participants were then read standardised think aloud instructions adapted from Green and Gilhooly [39] and French, Cooke, McLean, Williams and Sutton [40]. The researcher answered any questions before starting the recording. Next, participants were given a practise task to familiarise themselves with the think aloud process. They completed five randomly selected items of the Me and My School Questionnaire (MMSQ) [41]. The MMSQ was chosen as it has a similar response format to the five questionnaires used in this study. It has also been validated for adolescent use [41]. The participants then completed the think aloud process for the remaining questionnaires.

Power relations, informed consent and confidentiality are the main ethical considerations when interviewing minors [42]. As informed consent was provided by a parent/guardian, assent by the participant, and confidentiality including anonymity assured, to minimise the power relation and influence, when the interview was conducted in person the researcher sat outside the line of sight of the participant. If the participant stopped talking for more than 10 seconds, the researcher told them to “please keep talking”. On average, interviews took 8 minutes 18 seconds (SD = 1 minute 38 seconds). Each transcript was transcribed verbatim.

### Materials and measures

Self-report measures were selected to align with Bracey’s [19] definitions of psychological well- and ill-being. To ensure consistency throughout, participants were asked to answer the questionnaires based on their feelings in their general life over the past week.

#### Positive and Negative Affect.

The 10-item Positive and Negative Affect Scale-Child [25] has five positive affect items (e.g., “happy” and “cheerful”) and five negative affect items (e.g., “sad” and “mad”) which participants rated from 1 (never) to 5 (always).

#### Vitality.

A 5-item version of the Subjective Vitality Scale [26] was used to measure feelings of psychological energy and vitality. Items such as “I felt I had a lot of energy” and “I looked forward to each day” were rated from 1 (strongly disagree) to 5 (strongly agree).

#### Serenity.

All nine items from the inner haven subscale of the Brief Serenity Scale [28] were used to measure participants’ serenity. The items (e.g., “I am aware of inner peace” and “when I get upset, I become peaceful by getting in touch with my inner self”) were rated from 1 (never) to 5 (always).

#### Lethargy.

Where possible, the lethargy scale (LS) items were developed based on responses from participants in Bracey’s [19] study, addressing a key critique that previous measures were created without their involvement. For example, a 15-year-old boy said “sluggish…not want to do anything” [19] which became the items “I was tired a lot” and “I had no energy for things”. Four items were rated from 1 (never) to 5 (always).

### Data analysis

Descriptive statistics (means and standard deviations) were calculated using IBM SPSS Statistics (Version 29) [35] to examine the similarity of responses to previous research findings to ensure the think aloud process did not impact how participants answered the questionnaire. The audio recordings were transcribed by the first author. The think aloud transcripts were then thematically analysed using Braun and Clarke’s [43] six-phase guide. The six steps are: (1) familiarizing yourself with your data, (2) generating initial codes, (3) searching for themes, (4) reviewing themes, (5) defining and naming themes, and (6) producing the report. Each item was assigned to one of two themes: no problem, or response problem. The response problem theme consisted of four sub-themes: stumbled or reread item, clarified understanding of item, misinterpreted item, and did not understand item. These themes were adapted from French et al. [40]. An item was reviewed if it fell into any of the response problem sub-themes across five or more participants. Finally, the reading age of the reviewed questionnaires were calculated using the Flesch-Kincaid Reading Grade Level (FKRGL) [44] in Microsoft Word. The FKRGL is determined by total number of words, total number of sentences, and total number of syllables [44]. A lower score denotes a lower reading age.

## Results

### Descriptive statistics

The ethnicity of participants is shown in Table 1. The most common ethnicity was White – British (37.5%) followed by Black – African and Mixed – White and Black Caribbean (12.5% each). Table 2 shows the descriptive statistics for each questionnaire. Any participants who answered “prefer not to answer” were excluded from the analysis of that item. All well-being scores were above the mid-point indicating moderately high levels, and all ill-being scores were below the midpoint indicating moderately low levels.

**Table 1:** Ethnicity of participants (Sample 1).

Ethnicity	%
Asian or Asian British – Indian	10
Asian or Asian British – Pakistani	2.5
Asian or Asian British – Bangladeshi	2.5
Black – Caribbean	10
Black – African	12.5
Mixed – White and Black Caribbean	12.5
Mixed – White and Black African	2.5
Other	0
White – British	37.5
White - Other	10

**Table 2:** Descriptive statistics for each subscale (Sample 1).

Scale	Minimum-Maximum	Number of Participants	Mean Score	Standard Deviation
*Well-Being*				
PANAS-C (PA items)	1-5	40	3.70	0.99
SVS	1-5	40	3.66	1.01
BSS	1-5	38	3.52	1.03
*Ill-Being*				
PANAS-C (NA items)	1-5	39	1.93	0.94
LS	1-5	39	2.50	1.01

BSS = Brief Serenity Scale; LS = Lethargy Scale; NA = Negative Affect; PA = Positive Affect; PANAS-C = Positive and Negative Affect Scale – Child Form; SVS = Subjective Vitality Scale.

### Thematic analysis

Table 3 shows the number of problems experienced by participants for each questionnaire. A ‘problem’ was an item coded under any of the response problem sub-themes in the thematic analysis. An item was deemed a ‘problematic item’ if this happened across five or more participants. On average, participants experienced 4.4 problems when completing the questionnaires. Only two participants experienced no problems. PANAS-C had a total of five problems across five items, LS had a total of 60 problems across four items, SVS had a total of 13 problems across five items, and the BSS had the most problems with a total of 122 across nine items.

**Table 3:** Number of problems for each subscale (Sample 1).

Scale	Total number of problems	Number of problems per coding category				
		1	2	3	4	5
PANAS-C	5	395	0	2	0	3
LS	60	125	26	1	2	31
SVS	13	188	10	1	0	2
BSS	122	258	42	6	3	72

BSS = Brief Serenity Scale; LS = Lethargy Scale; PANAS-C = Positive and Negative Affect Scale – Child Form; SVS = Subjective Vitality Scale.

1 = no significant problems identified; 2 = participant stumbled or reread item; 3 = participant clarified understanding of item; 4 = participant misinterpreted item; 5 = participant did not understand item.

Table 4 shows the number of participants experiencing problems per item. Problematic items are shown in bold. Nine out of 28 items had no problems. The item with the most problems was “I felt lethargic” from the LS with 58 problems experienced across 33 participants.

**Table 4:** Number and type of problem for each item (Sample 1).

Item	Total number of problems	Number of problems per coding category				
		1	2	3	4	5
*PANAS-C*						
Cheerful	0	40	0	0	0	0
Lively	3	37	0	1	0	2
Happy	0	40	0	0	0	0
Joyful	1	39	0	1	0	0
Proud	1	39	0	0	0	1
Miserable	0	40	0	0	0	0
Mad	0	40	0	0	0	0
Afraid	0	40	0	0	0	0
Scared	0	40	0	0	0	0
Sad	0	40	0	0	0	0
*LS*						
**I felt lethargic.**	**33**	**7**	**25**	**1**	**1**	**31**
I had no energy for things.	1	39	1	0	0	0
I was tired a lot.	0	40	0	0	0	0
I was excited to do things. (R)	1	39	0	0	1	0
*SVS*						
I felt full of excitement.	0	40	0	0	0	0
I had high spirits.	2	38	0	1	0	1
I looked forward to each day.	2	38	2	0	0	0
**I nearly always felt alert and awake.**	**6**	**34**	**6**	**0**	**0**	**1**
I felt I had a lot of energy.	2	38	2	0	0	0
*BSS*						
**I feel serene.**	**37**	**3**	**18**	**3**	**0**	**34**
**I’m aware of an inner source of comfort, strength and security.**	**7**	**33**	**2**	**0**	**1**	**5**
**During troubled time I experience an inner source of strength.**	**9**	**31**	**6**	**0**	**0**	**3**
**I experience peace of mind.**	**6**	**34**	**2**	**2**	**0**	**2**
**I am aware of inner peace.**	**8**	**32**	**0**	**2**	**0**	**6**
**I experience an inner quiet that does not depend on events.**	**17**	**23**	**2**	**0**	**1**	**14**
**When I get upset, I become peaceful by getting in touch with my inner self.**	**8**	**32**	**5**	**0**	**0**	**3**
I experience an inner calm even when I’m under pressure.	4	36	2	0	0	2
**I can feel angry and observe my feelings of anger and separate myself from it and still feel an inner peace.**	**6**	**34**	**4**	**0**	**0**	**3**

Bold = problematic item.

BSS = Brief Serenity Scale; LS = Lethargy Scale; PANAS-C = Positive and Negative Affect Scale – Child Form; R = Reverse Scored; SVS = Subjective Vitality Scale.

1 = no significant problems identified; 2 = participant stumbled or reread item; 3 = participant clarified understanding of item; 4 = participant misinterpreted item; 5 = participant did not understand item.

### Items for review

Items deemed problematic following thematic analysis were reviewed. No items from the PANAS-C needed to be reviewed. One item from the LS, one item from the SVS, and eight items from the BSS were reviewed.

#### Lethargy Scale.

For the item “I felt lethargic”, 58 problems were experienced across 33 participants. Thirty-one participants explicitly stated that they did not understand what “lethargic” meant. Of these 31 participants, 14 struggled to, or incorrectly pronounced lethargic, and nine participants did not attempt to pronounce “lethargic” before stating they did not know what it meant. These participants opted to point to the item and ask for help. For example, [points to lethargic] “what does that mean?”. The researcher responded by saying “say it out loud, and if you still don’t understand it, tell me”. The participant attempted but still could not pronounce lethargic: “lef..ar… I don’t know” (P7). Participant 37 misunderstood the meaning of lethargic saying “yeah, like sick” after being asked to clarify their understanding of the item by the researcher. Finally, one participant clarified their understanding before answering the item: “what is that, tired?” (P24).

#### Subjective Vitality Scale.

Six participants had a problem with the item “I nearly always felt alert and awake”. All six participants stumbled or reread the item. For example, “I nearly fell…wait I nearly always felt alert and awake” (P38). Only one participant did not understand the item, “I nearly always felt awake…alert and awake. I don’t understand” (P12).

#### Brief Serenity Scale.

The most problematic item was “I feel serene”, as 36 participants experienced at least one problem with it. Of these 36 participants, 33 did not know what the word “serene” meant, and three participants had to clarify their understanding of serene. Responses included “I dunno what serene is” (P3) and “what’s serene? Calm?” (P21). Moreover, 18 participants stumbled or had to reread the item. The difficulty in pronouncing the word “serene” is demonstrated by participant 1 “I feel sincere. Is that sincere?” and participant 31 “I feel sincere. What does that mean?”.

Seven participants had problems with the item “I’m aware of an inner source of comfort, strength and security”. Five participants did not understand the item, such as participant 7 who said “I’m aware of an inner…inner source of comfort, strength and security. Uh I dunno”. Participant 4 also stumbled and reread the item as they said: “I’m aware of inner source…I am aware of an inner source of comfort, strength and security”. Lastly, one participant misunderstood the item, as they focused only on the “comfort” aspect of the item, neglecting “strength and security” from their thought process for their answer. They asked “what does that mean? … does that mean like I have my comforts and that sort of stuff?” (P18).

The item “during troubled times I experience an inner source of strength” was problematic for seven participants, with a total of nine problems. Three participants did not understand the item, for example “what? I don’t understand” (P12). Six participants stumbled or reread the question, such as “during trouble…troubled times I…I experience an inner source of strength” (P7) and “during troubled timed. Wait, during troubled times I experience an inner source of strength” (P9).

There was a total of six problems for the item “I experience peace of mind”. Two participants had to clarify their understanding, two participants stumbled or had to reread the item, and two participants did not understand the item. Example quotes include “umm. I experience peace of mind. What’s that? Like peace *in* your mind?” (P1), “I experience peace of mind…hmm… wait… not sure” (P5), and “I experience peace of mind. Does that mean like peace?” (P12).

Eight participants had a problem with the item “I am aware of inner peace”. Six of these participants did not understand the item, including confusion regarding what “inner peace” meant such as “what’s inner peace?” (P30). Two participants had to clarify their understanding of the item. For example, participant 22 said “what does it mean by aware, because like I know it, but I don’t have it… well I am aware that I have it because if someone says sorry then I’m like peace”. Participant 10 clarified their understanding by asking “does that mean like peaceful on the inside?”.

Another problematic item was “I experience an inner quiet that does not depend on events”. A total of 17 participants experienced a problem, with two participants rereading the item, one participant misinterpreting the item, and 14 participants not understanding the item. Problems occurred due to both the “inner quiet” aspect and the “does not depend on events” aspect of the item. For example, “does that mean your body is like quiet?” (P18) and “the end bit. Like the inner quiet, that’s what’s happening in your mind. But the end bit” (P31).

Three participants did not understand the item “when I get upset, I become peaceful by getting in touch with my inner self”, and five participants stumbled or had to reread the item. For example, participant 1 stumbled by saying “and when I’m…when I get upset, I become peaceful and get in touch with my inner self.” Participant 12 read the item correctly and then said “no, don’t understand”. Similarly, participant 28 had difficulty as they said, “what does that mean?”.

Finally, seven problems occurred across six participants with the item “I can feel angry and observe my feelings of anger and separate myself from it and still feel an inner peace”. Three problems were due to not understanding the item. For example, “These questions! I don’t know” (P12). Four problems were due to stumbling or rereading the item, as shown by participant 4 who said, “I feel angry and observe…I feel…I can feel angry and observe my feelings of anger and separate myself from it and still feel...” and answered “never” without reading the item in full. Participant 9 stumbled, reread the question, and still did not understand it. They said “wait I need to read that again… I can feel angry and observe my feelings of anger and sep-ar-at myself, separate myself from it and feel and still feel an inner peace.” This was followed by a 3 second pause, then they looked at researcher for help.

### Changes to items

The items that required changes are shown in Table 5. Initially participants’ suggestions during the think aloud were used to reword problematic items so they could be more easily understood by young people. Some items however were deemed unsuitable and so were removed.

**Table 5:** Changes made to items for review (Sample 1).

Scale	Item	Change Made
LS	I felt lethargic.	Remove item.
SVS	I nearly always felt alert and awake.	I mostly felt alert and awake.
BSS	I feel serene.	Remove item.
BSS	I’m aware of an inner source of comfort, strength and security.	Remove item.
BSS	During troubled time I experience an inner source of strength.	Remove item.
BSS	I experience peace of mind.	Remove item.
BSS	I am aware of inner peace.	Remove item.
BSS	I experience an inner quiet that does not depend on events.	Remove item.
BSS	When I get upset, I become peaceful by getting in touch with my inner self.	Remove item.
BSS	I can feel angry and observe my feelings of anger and separate myself from it and still feel an inner peace.	Remove item.

BSS = Brief Serenity Scale; LS = Lethargy Scale; SVS = Subjective Vitality Scale.

#### Lethargy Scale.

It was decided to remove the item “I felt lethargic”. Although participant 4 provided a suitable rewording, “what is that, tired?”, this was too similar to the item “I felt tired a lot”.

#### Subjective Vitality Scale.

Participants seemed to stumble on the “nearly always” part of the item “I nearly always felt alert and awake”. After asking the six participants that stumbled on “almost always” to reword the item, the most frequent (N = 4) suggestion was “mostly”. So, the item was reworded based on this participant feedback to “I mostly felt alert and awake”.

#### Brief Serenity Scale.

The suggestions from the participants on rewording the BSS items were discussed and considered by the study’s authors. However, after this process, the original and reworded items were deemed by the researchers as too complex for adolescents. The decision was therefore made to search for an alternative serenity scale whose items fit the definition of serenity used for this study, and were less challenging for young people to read and understand. This is described in the section below.

## Revised measure of serenity

### Materials

The search for an alternative serenity scale revealed the Child Serenity Scale (CSS) [45] which has 6-items measured on a 3-point Likert scale of “yes”, “more or less”, and “no”. The CSS was originally written in Spanish and developed in Argentina with Argentine children. Although items were available in English [45], it was decided to translate the original Spanish items into English, and then translate them back into English with the assistance of two bilingual researchers. The aim of the CSS is to determine a child’s ability to “regulate emotion and their responses to stressful events” [45]. Although this differs from Bracey’s [19] definition of serenity (“a positive state of deactivation marked by composed tranquillity and inner peace”), the items (e.g., “I am often a calm person”) reflect Bracey’s definition making it, according to COSMIN guidelines [23], a relevant and comprehensive measure of adolescents’ serenity.

### Procedure

The same procedures and method described above were used for the CSS [44] with 57 new participants (M = 13.2, SD = 0.38) who completed a think aloud interview in person. Participants were females aged 13 or 14 years who live in the UK. Data collection started on 12/06/2024 and ended on 05/07/2024.

### Data analysis

Descriptive statistics were calculated using SPSS [35]. Thematic analysis was conducted using the same protocol as the first think aloud. On average interviews took 4 minutes 23 seconds (SD = 42 seconds).

## Results

### Descriptive statistics

Table 6 shows the ethnicity of the participants. The most common ethnicity was Asian or Asian British – Indian (36.7%) followed by White – British (19.3%). Table 7 shows the descriptive statistics for the CSS. Any participants who answered “prefer not to answer” were excluded from the analysis of that item. Participants averaged a score of 2.26 out of 3, reflecting high levels of serenity.

**Table 6:** Ethnicity of participants (Sample 2).

Ethnicity	%
Asian or Asian British – Bangladeshi	1.8
Asian or Asian British – Pakistani	7.0
Asian or Asian British – Indian	36.7
Black – Caribbean	5.3
Black – African	10.5
Mixed – White and Black Caribbean	8.8
Other	1.8
White – British	19.3
White - Other	8.8

**Table 7:** Descriptive statistics of the Child Serenity Scale (Sample 2).

Scale	Minimum-Maximum	Number of Participants	Mean Score	Standard Deviation
CSS	1-3	57	2.26	0.58

CSS = Child Serenity Scale.

### Thematic analysis

Table 8 shows the total number of problems experienced by participants completing the CSS. On average, participants experienced 0.28 problems when completing the questionnaire. A total of 16 problems were experienced. Forty-four participants experienced no problems.

**Table 8:** Number of problems for the Child Serenity Scale (Sample 2).

Scale	Total number of problems	Number of problems per coding category				
		1	2	3	4	5
CSS	16	326	16	0	0	0

CSS = Child Serenity Scale.

1 = no significant problems identified; 2 = participant stumbled or reread item; 3 = participant clarified understanding of item; 4 = participant misinterpreted item; 5 = participant did not understand item.

Table 9 shows the number of problems per item. “Most days there are times when I feel peaceful” and “I stay calm even if I can’t do what I like” had the most problems, both with four participants rereading the item. One participant reread the item “I am often a calm person” before answering. Two participants stumbled or reread “I solve my problems very calmly”. For example “I solve…I sol..I solve my problems very calmly” (P6). Two participants stumbled or reread “I often feel relaxed” as shown by participant 9, “I feel… no I often feel relaxed”. Four participants stumbled or misread “most days there are times when I feel peaceful”. For example, “most days are...most days there are times when I feel peaceful” (P10). Three participants reread the item “even if I have a problem, I can stay calm”. Four participants stumbled or misread “I stay calm even if I can’t do what I like”. For example “even if I…I stay calm even if I can’t do what I like” (P7). As no item had problems across five or more participants, all items were deemed suitable to use with adolescent females aged 13–14 years old, and no changes needed to be made.

**Table 9:** Number and type of problem for each item (Sample 2).

Item	Total number of problems	Number of problems per coding category				
		1	2	3	4	5
*CSS*						
I am often a calm person.	1	56	1	0	0	0
I solve my problems very calmly.	2	55	2	0	0	0
I often feel relaxed.	2	55	2	0	0	0
Most days there are times when I feel peaceful.	4	53	4	0	0	0
Even if I have a problem, I can stay calm.	3	54	3	0	0	0
I stay calm even if I can’t do what I like.	4	53	4	0	0	0

CSS = Child Serenity Scale.

1 = no significant problems identified; 2 = participant stumbled or reread item; 3 = participant clarified understanding of item; 4 = participant misinterpreted item; 5 = participant did not understand item.

### Overall retained items

Table 10 shows the number of items retained, modified or removed from each sample. Out of the 28 items across questionnaires in Sample 1, 18 items were retained overall of which one item was modified and 9 items were removed. In Sample 2, all 6 items were retained without modifications. The resultant 24 items are shown in Table 11.

**Table 10:** Number of items retained, changed or removed from each sample.

Number of items tested	Number of items retained		Number of items removed
	Without changing	After changing	
*Sample 1*			
*27*	17	1	9
*Sample 2*			
6	6	0	0
*Total*			
33	23	1	9

**Table 11:** Resultant 24-item psychological well- and ill-being self-report measure.

Below are some statements about feelings. Please rate how you have felt this way over the *past week.*	(1) Never	(2) Rarely	(3) Sometimes	(4) Very Often	(5) Always	Prefer not to answer
1. I felt cheerful.						
2. I felt lively.						
3. I felt happy.						
4. I felt joyful.						
5. I felt proud.						
6. I felt miserable.						
7. I felt mad.						
8. I felt afraid.						
9. I felt scared.						
10. I felt sad.						
11. I had no energy for things.						
12. I was tired a lot.						
13. I was excited to do things.						
14. I felt full of excitement.						
15. I had high spirits.						
16. I looked forward to each day.						
17. I mostly felt alert and awake.						
18. I felt I had a lot a lot of energy.						
19. I was a calm person.						
20. I solved my problems very calmly.						
21. I felt relaxed.						
22. Most days there were times when I felt peaceful.						
23. Even if I had a problem, I stayed calm.						
24. I stayed calm even if I couldn’t do what I like.						

### Reading age

The reading age for the resultant 24 items was 1.1 on the Flesch-Kincaid Reading Grade Level [44]; this is equivalent to five years old.

### Response scale

The questionnaires used in this study have different response scales. This caused some confusion amongst participants; not all participants understood what “neutral” meant which is used by the SVS. There were no problems experienced by any participant with the ratings “never”, “rarely”, “sometimes”, “very often”, and “always” as used by the BSS and PANAS-C.

## Discussion

Adolescents have previously defined psychological well-being as having life satisfaction, happiness, and optimal functioning characterised by positive affect, vitality, and serenity and psychological ill-being as negative emotional states and undesirable psychological conditions determined by negative affect, and lethargy [19]. These definitions reveal the multidimensional nature of psychological well- and ill-being as experienced by young people. Building upon Bracey’s [19] initial work, the purpose of this study was to examine the content validity of questionnaire measures of the aforementioned indicators of psychological well- and ill-being using think aloud methods with a sample of 13–14-year-old girls. When testing the content validity of a self-report measure, COSMIN guidelines [23] state items need to be relevant, comprehensive, and comprehendible by the researchers and participants. Selecting items based on Bracey’s [19] indicators of psychological well- and ill-being ensured that the content was relevant and comprehensive. The think aloud interviews would reveal whether the items were comprehendible by the participants.

Descriptive statistics were initially calculated to assess the similarity of the participants’ psychological well- and ill-being scores to those reported in previous research. In this study, on average, participants scored 3.7 out of five for positive affect and 1.93 out of five for negative affect which is similar to mean scores (M = 3.38 and M = 1.80) reported in the original work of Ebesutani et al. [25]. Likewise, participants’ mean score for vitality in this study was 3.66 out of five. This aligns with previous use of SVS [26] where adolescent football players aged 10–14 years old in the UK had a mean score of 3.98 [27]. In this study, participants scored an average of 2.26 out of three for serenity using the CSS, which is similar to participants in Argentina aged 9–13 years old who had a mean score of 2.18 [46]. Finally, participants reported a mean score of 2.50 out of five for lethargy in this study, but due to the lack of adolescent lethargy measures, no comparison can be made. The similarity of the scores in this study compared to previous research suggests the think aloud interview did not impact the participants’ scoring.

Thematic analysis was used to code transcripts and create themes based on the French et al. [40] protocol. There were two main themes: no problem and response problem. The response problem theme was split into four sub-themes: stumbled or reread item, clarified understanding of item, misinterpreted item, and did not understand item. An item was deemed problematic and reviewed if it fell into the response problem theme across five or more participants. No items from the PANAS-C [25] needed to be reviewed. One item of the SVS [26], one item from the LS, and eight items from the BSS inner haven subscale [29] were reviewed.

For the SVS [26] the item “I nearly always felt alert and awake” was changed to “I mostly felt alert and awake” based on feedback from the participants. For the LS, the item “I felt lethargic” was removed because participants’ suggestions to reword the item to “I felt tired”, “I didn’t want to do anything” or “I had low energy” were too similar to other items on the lethargy scale.

The analysis revealed significantly more issues with the BSS items. Even after considering suggestions from the participants, and discussions from the four researchers how to reword the items, eight items were deemed too complex for adolescents. These problems may have occurred due to the linguistic features of the items, such as multi-clause sentences (e.g., “I can feel angry and observe my feeling of anger and separate myself from it and still feel an inner peace”), and complex abstract language (e.g., “inner self”, “inner calm”, and “inner peace”). When developing a questionnaire for adolescents, items should be unambiguous and simple, avoiding double-barrelled questions [47]. This is in line with COSMIN guidelines where comprehensibility is a key element of item development [23].

Subsequently, the Child Serenity Scale (CSS) [45] was identified and back translated from Spanish to English, then tested using the same think aloud protocol with a second sample of adolescent girls. Data analysis revealed no problems with the six items of the CSS. Items were most likely understood by the participants due to the simplicity of items (e.g., “I was a calm person”).

The removal of problematic items across the questionnaires resulted in 24 items, 16 measuring well-being and eight measuring ill-being (Table 11), which have a suitable reading age (1.1 on the FKRGL [44]) for the target population of 13–14-year-olds. Even though the BSS had a suitable reading age for 13–14 year olds (5.2 equivalent to 11 years old), participants still identified problems with the items. Therefore, it was important that the final items had a suitable reading age, and simple linguistic features such as no complex abstract (e.g., “inner”) feelings that young people may be less familiar with.

The results of the thematic analysis confirm the importance of including the target population during the development and validation of a questionnaire. The PANAS-C [25] and the CSS [45] were the only scales where children or adolescents were involved in the original development of the measure, and in this study, were the only scales where no changes were needed. The results also highlight that existing adult measures of psychological well- and ill-being may not produce valid or reliable results when used with adolescents, due to younger people not understanding all items of the measure. Future studies should seek to use measures that were developed with younger people’s input to reflect their current understanding and experience of psychological well- and ill-being, and with their reading age in mind.

### Limitations

A limitation of this study was having to create the items to measure lethargy, rather than using an existing questionnaire. However as mentioned, no measure that fits the definition of lethargy used in this study exists, and the items were worded based on adolescent feedback from Bracey’s [19] work. The results also confirmed the sample understood the retained three items. Another limitation is that the study only focuses on one type of validity (construct). Future research should test other forms of validity (e.g., using factor analysis), invariance, and the reliability of the scales used in this study and/or the identified 24 items from the think aloud interviews. This would further strengthen the confidence in using the scales or items with adolescents. A further limitation is that neither the academic performance nor the reading skill of participants were measured. However, as these participants were recruited from highly deprived areas (which is an indicator of reading skill [48], it may be expected that other female adolescents from less deprived areas will understand the items. This is something that future research should determine. Moreover, only female participants aged 13–14 years from the UK were recruited. This study focused on teenage girls s because this population is particularly vulnerable to lower well-being and higher ill-being levels [34]. However, it means the results cannot be generalised to other populations. Additionally, a small cluster of schools and sports clubs were used to recruit participants which also limits the generalisability of the results. Future research should aim to use think aloud with additional samples (e.g., adolescent boys; older adolescent girls; samples from other countries) to provide further evidence regarding the construct validity of the targeted questionnaires. Purposive sampling could be used to ensure participants from a variety of socioeconomic backgrounds and reading skill are also recruited.

## Conclusions

This think aloud study revealed female adolescents aged 13–14 years old did not understand some of the items on the targeted measures of psychological well- and ill-being, despite many of the measures being extensively used in research. By not understanding the items, adolescents may not be answering the questionnaires with content or construct reliability, therefore influencing their scores. This study proposes changes, suggested by female adolescents, that could be made to the wording of the items which are more suitable for this population, thus providing more accurate scores and reflections of their psychological well- and ill-being. If future studies further test and establish the psychometrics of the resultant 24-item questionnaire, it could be an important tool to measure adolescent psychological well- and ill-being both in research and in applied settings such as schools.

### Public significance statement

This study suggests that female adolescents aged 13–14 years do not understand all items on the psychological well- and ill-being self-report measures that were tested. It could be argued that previous research conducted among adolescents in this age group using these measures might not be reliable or valid. Rewording problematic items based on participant feedback resulted in a 24-item self-report measure that future research should seek to validate.

## Supporting information

S1 File Questionnaire pack provided to participants for part 1 and part 2 of the study. These are the original items before amendments were suggested.
(DOCX)
