# Supplementary material for: Using think aloud with female adolescents to validate psychological well- and ill-being self-report measures
Source: PLOS Ment Health. 2026 Feb 17;3(2):e0000551. doi: 10.1371/journal.pmen.0000551 (PMC12912544; doi:10.1371/journal.pmen.0000551)
Supplement: S1 File — These are the original items before amendments were suggested. (DOCX) [file pmen.0000551.s001.docx]

**VALIDATING ADOLESCENT WELL- AND ILL-BEING MEASURES**

***Part 1***

We would like to know more about your feelings. There are no right or wrong answers, so please answer honestly. Your answers will be anonymous meaning no one other than yourself will know how you have responded. Whilst we would like you to respond to all the statements, you can choose not to answer any you do not want to.

| Below are a number of statements relating to feelings and your everyday life. Please rate how you have felt this way over the ***past week****.* | | | | | | |
| --- | --- | --- | --- | --- | --- | --- |
|  | **Never** | **Rarely** | **Sometimes** | **Very often** | **Always** | **Prefer not to answer** |
| 1. I felt cheerful. | 1 | 2 | 3 | 4 | 5 | X |
| 2. I felt lively. | 1 | 2 | 3 | 4 | 5 | X |
| 3. I felt happy. | 1 | 2 | 3 | 4 | 5 | X |
| 4. I felt joyful. | 1 | 2 | 3 | 4 | 5 | X |
| 5. I felt proud. | 1 | 2 | 3 | 4 | 5 | X |
| 6. I felt miserable. | 1 | 2 | 3 | 4 | 5 | X |
| 7. I felt mad. | 1 | 2 | 3 | 4 | 5 | X |
| 8. I felt afraid. | 1 | 2 | 3 | 4 | 5 | X |
| 9. I felt scared. | 1 | 2 | 3 | 4 | 5 | X |
| 10. I felt sad. | 1 | 2 | 3 | 4 | 5 | X |

| Below are a number of statements relating to lethargy and your everyday life. Please rate how you have felt this way over the ***past week****.* | | | | | | |
| --- | --- | --- | --- | --- | --- | --- |
|  | **Never** | **Rarely** | **Sometimes** | **Very often** | **Always** | **Prefer not to answer** |
| 1. I felt lethargic. | 1 | 2 | 3 | 4 | 5 | X |
| 2. I had no energy for things. | 1 | 2 | 3 | 4 | 5 | X |
| 3. I was tired a lot. | 1 | 2 | 3 | 4 | 5 | X |
| 4. I was excited to do things. | 1 | 2 | 3 | 4 | 5 | X |

| Below are a number of statements relating to vitality your everyday life. Please rate the extent to which you agree or disagree with the statement over the ***past week****.* | | | | | | |
| --- | --- | --- | --- | --- | --- | --- |
|  | **Strongly Disagree** | **Disagree** | **Neutral** | **Agree** | **Strongly**  **Agree** | **Prefer not to answer** |
| 1. I felt full of excitement. | 1 | 2 | 3 | 4 | 5 | X |
| 2. I had high spirits. | 1 | 2 | 3 | 4 | 5 | X |
| 3. I looked forward to each day. | 1 | 2 | 3 | 4 | 5 | X |
| 4. I nearly always felt alert and awake. | 1 | 2 | 3 | 4 | 5 | X |
| 5. I felt I had a lot of energy. | 1 | 2 | 3 | 4 | 5 | X |

| Below are a number of statements relating to serenity and your everyday life. Please rate how you have felt this way over the ***past week****.* | | | | | | |
| --- | --- | --- | --- | --- | --- | --- |
|  | **Never** | **Rarely** | **Sometimes** | **Very often** | **Always** | **Prefer not to answer** |
| 1. I feel serene. | 1 | 2 | 3 | 4 | 5 | X |
| 2. I am aware of an inner source of comfort, strength, and security. | 1 | 2 | 3 | 4 | 5 | X |
| 3. During troubled times, I experience an inner source of strength. | 1 | 2 | 3 | 4 | 5 | X |
| 4. I experience peace of mind. | 1 | 2 | 3 | 4 | 5 | X |
| 5. I am aware of inner peace. | 1 | 2 | 3 | 4 | 5 | X |
| 6. I experience an inner quiet that does not depend on events. | 1 | 2 | 3 | 4 | 5 | X |
| 7. When I get upset, I become peaceful by getting in touch with my inner self. | 1 | 2 | 3 | 4 | 5 | X |
| 8. I experience an inner calm even when I am under pressure | 1 | 2 | 3 | 4 | 5 | X |
| 9. I can feel angry and observe my feeling of anger and separate myself from it and still feel an inner peace. | 1 | 2 | 3 | 4 | 5 | X |

This is the end of the questionnaire.

Thank you for taking part.

If you have found any of the questions stressful, you can find support here: <https://www.youngminds.org.uk/>

***Part 2***

We would like to know more about your feelings. There are no right or wrong answers, so please answer honestly. Your answers will be anonymous meaning no one other than yourself will know how you have responded. Whilst we would like you to respond to all the statements, you can choose not to answer any you do not want to.

| Below are a number of statements relating to serenity and your everyday life. Please rate the extent to which the statements are true over the ***past six months****.* | | | | |
| --- | --- | --- | --- | --- |
|  | **No** | **Sometimes** | **Yes** | **Prefer not to answer** |
| 1. I was a calm person. | 0 | 1 | 2 | X |
| 2. I solved my problems very calmly. | 0 | 1 | 2 | X |
| 3. I felt relaxed. | 0 | 1 | 2 | X |
| 4. Most days there were times when I felt peaceful. | 0 | 1 | 2 | X |
| 5. Even if I had a problem, I stayed calm. | 0 | 1 | 2 | X |

This is the end of the questionnaire.

Thank you for taking part.

If you have found any of the questions stressful, you can find support here: <https://www.youngminds.org.uk/>
